# Prehabilitation Strategies: Enhancing Surgical Resilience with a Focus on Nutritional Optimization and Multimodal Interventions

**DOI:** 10.1016/j.advnut.2025.100392

**Published:** 2025-02-15

**Authors:** Suriyaraj Shanmugasundaram Prema, Dhanraj Ganapathy, Deepankumar Shanmugamprema

**Affiliations:** 1 Department of Biotechnology, Rathinam Technical Campus, Rathinam Techzone, Coimbatore, Tamil Nadu, India; 2 Department of Research Analytics, Saveetha Dental College and Hospitals, Saveetha Institute of Medical and Technical Sciences, Saveetha University, Chennai, India

**Keywords:** prehabilitation, nutritional optimization, multimodal interventions, perioperative care, surgical outcomes, personalized medicine

## Abstract

Surgery imposes significant physiological and psychological stress, often leading to complications, delayed recovery, and prolonged hospital stays. Prehabilitation, a proactive strategy to optimize patients’ resilience before surgery, has emerged as a transformative approach in perioperative care. Nutritional prehabilitation specifically addresses metabolic dysregulation, muscle loss, and immune suppression caused by surgical stress. This review highlights the critical role of nutritional prehabilitation within a multimodal framework, integrating exercise, psychological support, and emerging technologies. Although some evidence supports the effectiveness of prehabilitation in enhancing functional outcomes and improvements in rates of complications and mortality, its implementation faces challenges such as resources, lack of standardized protocols, and variability across healthcare settings, highlighting the need for greater standardization. Physical training as part of prehabilitation also improves mood, fosters patient engagement, and instills a sense of control over the disease process. These psychosocial benefits, alongside enhanced patient-reported outcomes and qualitative measures, reflect the holistic value of prehabilitation. Emerging technologies, such as wearable devices and telemedicine, offer scalable and personalized solutions for delivering prehabilitation, particularly in resource-limited settings. Future research should prioritize refining protocols, exploring long-term outcomes, and addressing the unique needs of high-risk populations. By emphasizing a proactive approach to perioperative care, this review aims to highlight the potential of nutritional prehabilitation as a foundational component of multimodal strategies designed to optimize surgical resilience, empower patients, and transform surgical recovery into a proactive and patient-centered journey.

**Statement of significance**

This review provides a comprehensive synthesis of the latest evidence on nutritional prehabilitation, emphasizing its integration with physical and psychological interventions to optimize surgical outcomes. By addressing the role of emerging technologies and highlighting challenges such as health disparities and implementation barriers, this study outlines a clear path for advancing global standards in prehabilitation.

## Introduction

Surgery represents a significant physiological challenge for patients, often marked by a cascade of inflammatory and metabolic responses that can compromise recovery and long-term health outcomes [1,2]. The perioperative period, encompassing the time before, during, and after surgery, is a critical window during which patients face significant physiological and psychological challenges, making them particularly vulnerable to complications such as infections, muscle wasting, physical deconditioning, and delayed healing [3,4]. Historically, surgical preparation emphasized immediate preoperative care and postoperative recovery. However, the paradigm has shifted to include prehabilitation as a proactive measure [5]. This approach encompasses several primary components: exercise, nutritional optimization, psychological preparation, and incorporates strategies such as alcohol and smoking cessation, anemia management, inspiratory muscle training, and patient education, all of which are crucial for improving overall patient health and readiness for surgery. Nutrition plays a central role in bolstering metabolic reserves, enhancing immune function, and maintaining muscle mass, supporting the effectiveness of these strategies. Although traditional surgical outcomes, such as mortality, length of hospital stay, and Clavien–Dindo classification, remain important, there is growing recognition of additional outcomes that are equally significant. These include functional outcomes, patient-reported outcomes, return to work, physical performance, mental health indicators like depression and anxiety, and oncological outcomes, particularly in cancer surgery. This expanded focus allows for a more comprehensive evaluation of patient recovery and long-term well-being.

**FIGURE 1:**
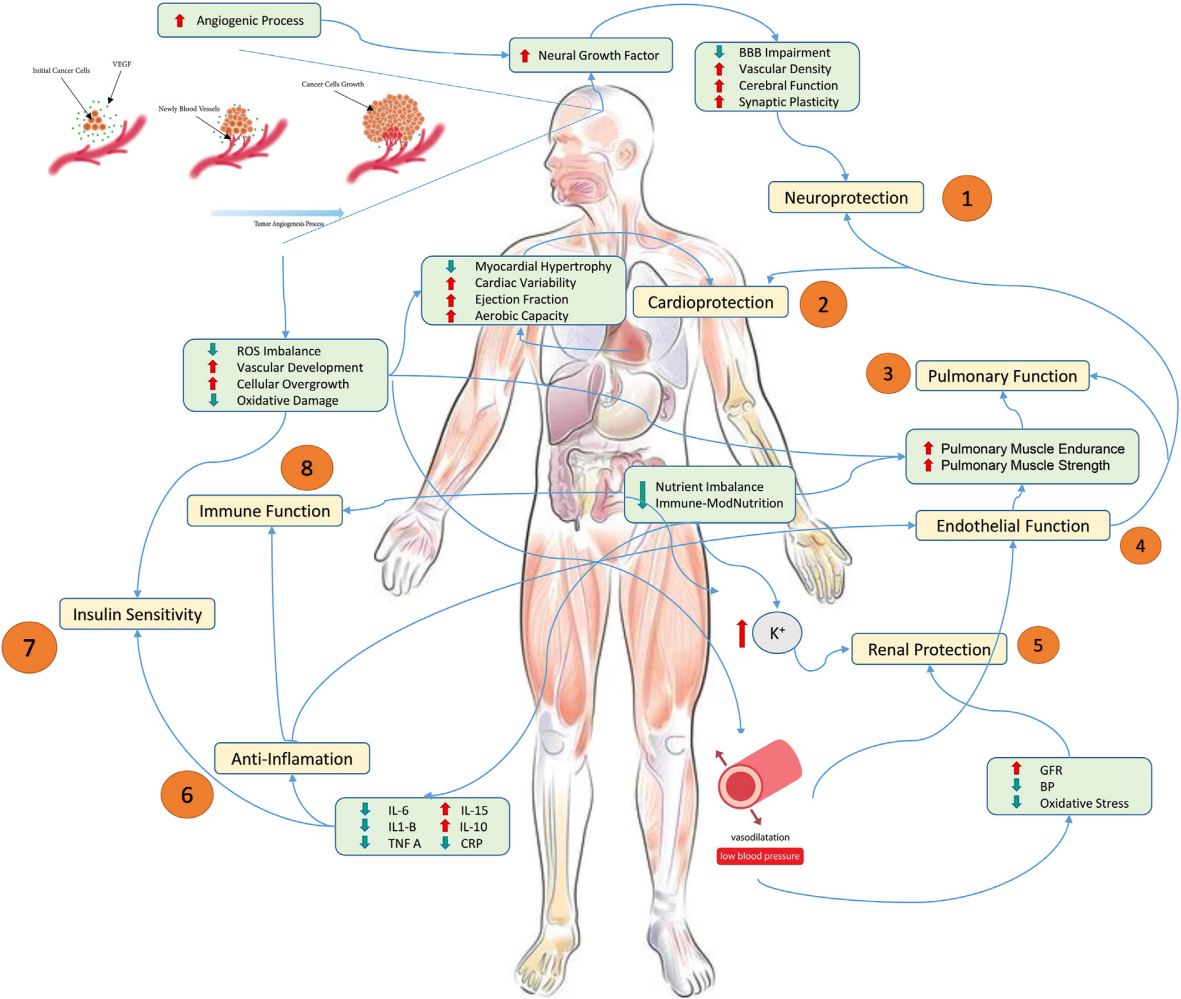
**Conceptual framework of prehabilitation strategies and outcomes.** Schematic illustrates the synergistic effects of nutritional, exercise, and psychological interventions in prehabilitation, emphasizing their role in enhancing physiological and psychological resilience before surgery. It highlights key pathways: *1*) neuroprotection, through stimulation of cerebral angiogenesis, increased cerebral perfusion, neuroplasticity, and maintenance of blood-brain barrier integrity; *2*) cardiopulmonary and musculoskeletal adaptations, driven by enhancements in protein synthesis, glucose metabolism, and oxidative remodeling; *3*) pulmonary function improvements, supported by increased surfactant production and strengthened respiratory muscles to enhance alveolar gas exchange; *4*) endothelial function, with benefits including vasoregulation, fibrinolysis, oxidative stress reduction, and anti-inflammatory signaling pathways; *5*) renal protection, achieved through reduced oxidative stress and improved glomerular filtration rate; *6*) inflammation regulation, targeting proinflammatory and immunosuppressive pathways; *7*) enhanced insulin sensitivity, mediated through cytokine signaling and improved glucose metabolism; and *8*) immune function maintenance, reducing risks of malnutrition, deconditioning, and psychological distress. Together, these interventions mitigate surgical stressors such as inflammation, oxidative stress, and immune suppression, facilitating faster recovery and reducing postoperative complications. BBB, Blood-Brain Barrier; BP, Blood Pressure; CRP, C-reactive protein; GFR, Glomerular Filtration Rate; IL, Interleukin; TNF-α, Tumor Necrosis Factor-Alpha; ROS, Reactive Oxygen Species; VEGF, Vascular Endothelial Growth Factor.

Malnutrition and poor nutritional status are prevalent among surgical patients, particularly those undergoing major cancer surgeries or gastrointestinal procedures. These patients often exhibit elevated inflammatory markers, depleted protein stores, and impaired immune responses [6,7]. Consequently, they face increased risks of postoperative complications and higher mortality rates. Nutritional status is a critical determinant of surgical outcomes, influencing immune function, wound healing, and muscle preservation. Malnutrition, even in its subclinical forms, significantly increases the risk of postoperative complications. It is estimated that ≤50% of patients undergoing major surgery are malnourished or at risk of malnutrition, underscoring the importance of nutritional optimization before surgery [8,9]. Nutritional prehabilitation seeks to correct deficits, build metabolic reserves, and enhance the patient’s ability to withstand surgical trauma. Nutritional prehabilitation offers a promising solution by addressing these deficits in the weeks leading up to surgery [10]. Surgery creates a significant metabolic burden, including inflammation, oxidative stress, and catabolic processes [11]. Surgical stress induces a state of hypermetabolism and insulin resistance, exacerbating protein catabolism and impairing glucose regulation. This metabolic response, although an adaptive mechanism for acute injury, can become pathological in the context of surgery, leading to muscle wasting, immunosuppression, and delayed recovery [12,13]. Nutritional interventions, such as high-protein diets, carbohydrate loading, and supplementation with specific micronutrients, aim to counteract these effects. By providing the necessary substrates for tissue repair and immune function, nutritional prehabilitation helps prevent significant functional decline, ensuring that the patient never falls below a critical threshold. This approach not only reduces risk of complications but also accelerates recovery, allowing the patient to actively engage in their recovery from medical, cognitive, psychological, physical, and functional perspectives [14]. The significance of prehabilitation extends beyond its physiological benefits (Figure 1).

Despite its promise, the implementation of prehabilitation faces several challenges. The progress in the field is hindered by a lack of standardization, with significant variability in the design, duration, and components of prehabilitation programs. The evidence base, although growing, remains fragmented, with studies often focusing on specific surgical populations or outcomes [15,16]. Moreover, resource constraints and systemic barriers, such as limited access to dietitians and other healthcare professionals involved in prehabilitation, hinder the widespread adoption of these programs. Addressing these issues requires a concerted effort to refine prehabilitation protocols, expand access, and generate high-quality evidence to support their efficacy. Emerging technologies offer new opportunities to overcome these barriers and enhance the delivery of prehabilitation. Wearable devices, telemedicine platforms, and artificial intelligence (AI)-driven tools are transforming the way prehabilitation is implemented and monitored [[17], [18], [19]]. These innovations enable personalized interventions, real-time tracking of progress, and greater accessibility for patients in remote or underserved areas. This manuscript explores the multifaceted dimensions of prehabilitation, with a focus on its nutritional aspects. It examines the physiological mechanisms through which nutritional prehabilitation enhances surgical outcomes, reviews the clinical evidence supporting its efficacy, and discusses its integration into multimodal frameworks. Additionally, it addresses the implementation challenges and future directions for research and practice in this field. By highlighting the critical role of nutrition in prehabilitation, this work aims to underscore its potential to transform perioperative care and improve the lives of patients undergoing surgery.

## Physiological Mechanisms of Nutritional Prehabilitation

Nutritional prehabilitation leverages targeted physiological mechanisms to enhance a patient’s capacity to withstand the metabolic and immunological challenges of surgery. Unlike generic dietary optimization, prehabilitation involves precise interventions to address the metabolic dysregulation induced by surgical trauma, including insulin resistance, systemic inflammation, protein catabolism, and immune suppression.

### Counteracting insulin resistance

Surgical stress triggers a pronounced increase in cortisol, catecholamines, and inflammatory cytokines, leading to insulin resistance. This state disrupts normal glucose homeostasis by impairing insulin-mediated glucose uptake, particularly in muscle and adipose tissue [20,21]. Instead, gluconeogenesis from amino acids becomes the primary energy source, exacerbating protein catabolism. Nutritional prehabilitation addresses insulin resistance primarily through preoperative carbohydrate loading. Providing a controlled amount of carbohydrates 2–3 h before surgery is hypothesized to stimulate insulin secretion, reducing the need for gluconeogenesis and preserving muscle protein stores [22]. Although evidence is still limited, improved glycemic control may help minimize hyperglycemia, which is strongly linked to postoperative complications such as infections and impaired wound healing. Additionally, enhancing insulin sensitivity could optimize cellular glucose uptake, maintaining the energy levels crucial for recovery.

### Modulating the inflammatory response

Surgical trauma activates a cascade of proinflammatory cytokines, including interleukin-6 (IL-6), tumor necrosis factor-alpha (TNF-α), and C-reactive protein. These cytokines induce systemic inflammation, impair immune function, and increase oxidative stress, which collectively delay healing and heighten risk of postoperative complications [23,24]. Immunonutrition plays a pivotal role in modulating this inflammatory response. Nutritional components such as arginine, omega-3 fatty acids, and glutamine are integral to this process. Arginine serves as a precursor for nitric oxide, which supports vasodilation, improves wound perfusion, and enhances macrophage-mediated pathogen clearance [25,26]. Omega-3 fatty acids, derived from fish oil, shift the inflammatory balance toward anti-inflammatory eicosanoids by competing with arachidonic acid in the cyclooxygenase pathway. This results in decreased production of proinflammatory mediators like prostaglandins and leukotrienes [27]. Glutamine supports lymphocyte proliferation and enhances intestinal barrier function, reducing systemic inflammation stemming from bacterial translocation. Micronutrients such as zinc, selenium, and vitamins A, C, and D play critical roles in supporting immune function during the perioperative period. Zinc acts as a cofactor for thymulin that regulates T-cell activity, thereby enhancing adaptive immunity [28,29].

### Preserving muscle protein stores

One of the most detrimental effects of surgical stress is the acceleration of muscle protein breakdown, driven by increased proteolysis and suppressed protein synthesis. This catabolic state arises from elevated glucocorticoid levels and systemic inflammation, which activate the ubiquitin–proteasome system and autophagy pathways in muscle cells [30,31]. Branched-chain amino acids (BCAAs), particularly leucine, stimulate the mechanistic target of rapamycin (mTOR) pathway, a key regulator of muscle protein synthesis [32,33]. By activating mTOR, leucine counteracts proteolysis and promotes anabolic signaling, preserving muscle mass and strength. Additionally, whey protein, rich in essential amino acids and BCAAs, is often included in prehabilitation protocols to enhance overall protein availability. Its rapid digestion, high bioavailability, and ability to deliver a large protein load to the bloodstream after ingestion make it particularly effective. When consumed 30–60 min after exercise, whey protein provides a significant anabolic stimulus, promoting the integration of nutritional protein into functional muscle. Adequate protein intake also supports the synthesis of acute-phase proteins, preserving the protein pool to better tolerate surgical stress and facilitating faster recovery of physical function postoperatively [34,35].

### Maintaining gut integrity and microbiome health

The gastrointestinal tract plays a central role in maintaining systemic homeostasis during surgical stress. However, surgical interventions can compromise gut barrier integrity, leading to increased permeability and bacterial translocation. This disruption exacerbates systemic inflammation and increases the risk of sepsis. Additionally, compromised gut integrity and inflammation can disturb iron homeostasis, leading to elevated levels of circulating free iron, which serves as a proseptic factor by promoting bacterial growth and exacerbating oxidative stress [36,37]. Although direct evidence in the perioperative setting is limited, prehabilitation strategies are hypothesized to support gut health through dietary fibers, prebiotics, and probiotics. Prebiotics, such as inulin and fructooligosaccharides, stimulate the growth of beneficial gut bacteria like *bifidobacteria*. Probiotic strains, including *Lactobacillus* and *Bifidobacterium*, are thought to enhance gut barrier integrity by increasing mucin production and tight-junction protein expression. These interventions may reduce systemic inflammation by limiting endotoxin translocation and modulating immune responses [38,39]. Glutamine supplementation, although not extensively studied in the perioperative context, has shown potential benefits in other clinical settings. As a primary energy source for enterocytes, glutamine is believed to enhance mucosal repair, reduce intestinal permeability, and prevent the activation of systemic inflammatory cascades. These mechanisms suggest it could contribute to improved surgical outcomes [40].

### Mitigating oxidative stress

Surgical stress generates excessive reactive oxygen species, which damage cellular components such as lipids, proteins, and DNA. Oxidative stress exacerbates inflammation, delays wound healing and impairs immune function [41]. Nutritional prehabilitation addresses this through antioxidant supplementation. Key antioxidants include vitamins C and E, selenium, and polyphenols. Vitamin E protects cell membranes from lipid peroxidation, although selenium, through its role in glutathione peroxidase, reduces hydrogen peroxide and organic hydroperoxides. Polyphenols, abundant in fruits and vegetables, are known to scavenge free radicals and enhance endogenous antioxidant enzyme activity. Evidence from in vitro studies suggests that these properties reduce oxidative stress, potentially creating a more favorable environment for healing and recovery [42].

## Clinical Evidence Supporting Nutritional Prehabilitation

The efficacy of nutritional prehabilitation has been explored across various clinical contexts, particularly in major surgical populations such as those undergoing cancer, gastrointestinal, and orthopedic procedures [43]. Evidence from randomized controlled trials (RCTs), systematic reviews, and meta-analyses has underscored its potential to enhance surgical outcomes (Table 1) [6,14,8,[44], [45], [46], [47], [48], [49], [50], [51], [52], [53], [54], [55]]. These findings highlight specific improvements in immune function, reduction in complications, preservation of muscle mass, and faster recovery of functional capacity [[56], [57], [58]]. However, the results have also revealed inconsistencies, which are largely attributable to heterogeneity in patient populations, intervention protocols, and study designs. Nutritional prehabilitation has shown significant promise in modulating the inflammatory response and improving immune function [59]. In surgical oncology, studies have demonstrated that immunonutrition can reduce the incidence of postoperative infections and sepsis. This finding has been supported by meta-analyses indicating that immunonutrition can reduce postoperative infection rates by as much as 30% in gastrointestinal surgeries. These improvements are particularly relevant in high-risk populations, such as patients with malnutrition or advanced age, who are more susceptible to inflammatory complications [60]. Muscle preservation is another critical outcome of nutritional prehabilitation, particularly in elderly patients and those with cancer cachexia. High-protein diets and amino acid supplementation have been shown to mitigate the muscle-wasting effects of surgical stress. A retrospective cohort study measured body composition using computed tomography (CT) scans before and after neoadjuvant chemotherapy in patients undergoing esophageal cancer surgery. The study found that prehabilitation with high-protein supplementation helped prevent the decline in functional and physical status during chemotherapy. However, no differences were observed between groups in postoperative outcomes, suggesting the intervention’s benefit lies in maintaining presurgical health rather than directly influencing surgical outcomes [61]. This effect was attributed to the anabolic properties of BCAAs, particularly leucine, which directly stimulate muscle protein synthesis via the mTOR signaling pathway. These findings align with broader evidence indicating that prehabilitation not only preserves muscle mass but also contributes to faster recovery of physical strength and mobility [62]. Functional recovery is a key focus of prehabilitation research, and nutritional interventions have been shown to enhance performance on objective measures such as the 6-min walk test (6MWT). In a study by Daniels et al. [63] involving patients undergoing major abdominal surgery, those who participated in a multimodal prehabilitation program that included nutritional support demonstrated significant improvements in functional capacity compared with those receiving standard care.

**TABLE 1:** Prehabilitation studies and outcomes.

Study	PMID	Country	Population	Outcome measurement	Intervention type	Time point	Prehab, *N*	Prehab, mean (SD)	Control, *N*	Control, mean (SD)	Effect size (95% CI)	Reported *P* value	Significance/conclusion
Barberan-Garcia et al. 2018 [44]	28489682	Spain	High-risk patients undergoing elective abdominal surgery	VO_2_ peak, postoperative complications	Multimodal (nutrition + high-intensity endurance training)	Presurgery	62	+135% ET gain (218)	63	No change in ET	Complications reduced: RR 0.5 (0.3, 0.8)	<0.001	Prehabilitation enhanced aerobic capacity, significantly reduced postoperative complications, and ICU stay length.
Gillis et al. 2016 [45]	26208743	Canada	Colorectal cancer surgery patients	Functional walking capacity (6MWT)	Nutrition counseling with whey protein supplementation	Pre- and post-op	22	+20.8 m (42.6)	21	+1.2 m (65.5)	Not significant	0.27	Nutrition prehabilitation with whey protein showed clinically meaningful improvement in presurgery functional capacity.
Walleret al., 2022 [46]	33723969	United Kingdom	Prehabilitation program patients	Adherence to walking regimens	Wearable activity tracking	Presurgery	11	+85.6 m (18.1–153.2)	11	+13.2 m (−6.8 to 33.2)	+72.4 (*P* = 0.014)	<0.05	Wearables significantly increase adherence to physical activity protocols.
Correia et al. 2019 [47]	30816849	Portugal	59 patients post-TKA (30 digital, 29 conventional)	Timed up and go (TUG), KOOS (symptoms, pain, ADL, sports, QoL), knee range of motion (ROM)	Digital biofeedback system compared with supervised home rehabilitation	Baseline, 3 mo, 6 mo	Digital: 30	TUG: 6.9 (1.6 sec), KOOS QoL: 94 (12.0)	Control: 29	TUG: 8.7 (4.0 s), KOOS QoL: 63 (37.5)	TUG median difference: 4.87 s (1.85, 7.47)	*P* < .001	Digital intervention showed superior outcomes across most measures, maintained ≤6 months.
Parker et al. 2021 [48]	33870744	United States	Patients in exercise program (EP) compared with in usual care (UC); localized patients with pancreatic cancer	Skeletal Muscle Index (SMI), skeletal muscle density	Home-based aerobic and resistance exercise compared with usual care	Baseline (T0) to preoperative restaging (T1)	33	SMI: 0.2 ± 3.2 cm^2^/m^2^	64	SMI: −1.4 ± 3.8 cm^2^/m^2^	SMI rate of change: +0.11 cm^2^/m^2^/wk (*P* = 0.02)	*P* = 0.03 for SMI difference between groups	Home-based exercise maintained SMI better than UC, suggesting improved skeletal muscle health during preoperative therapy.
Van Wijk et al. 2022 [49]	35851601	Netherlands	High-risk patients for liver/pancreatic resection	Aerobic fitness (VO_2_ at VAT and VO2peak)	Home-based bimodal prehabilitation (exercise + nutrition)	Presurgery	26	VO_2_ at VAT: +1.7 (1.1); VO2peak: +2.4 (1.4)	Not applicable	Not applicable	VO_2_ at VAT: +17.8%; VO2peak: +17.2%	VO_2_ at VAT: <0.001; VO2peak: 0.001	Significantly improves preoperative aerobic fitness in high-risk patients, with high adherence and satisfaction.
Chiu et al. 2023 [50]	37906193	Hong Kong	Adults undergoing elective surgery	Preoperative anxiety (APAIS)	Virtual reality (VR) + education	Pre- and post-op	37	Anxiety T2: 15.92 (4.67)	37	Anxiety T2: 20.59 (4.82)	–5.57 (–7.73, –3.41)	<0.001	VR intervention significantly reduced preoperative anxiety and stress although improving satisfaction and preparedness.
Minnella et al. 2018 [51]	30193337	Canada	Patients with esophagogastric cancer	Functional capacity, discharge time	Exercise + nutrition prehabilitation	Pre- and post-op	26	+36.9 m presurgery (51.4), +15.4 m postsurgery (65.6)	25	–22.8 m presurgery (52.5), –81.8 m postsurgery (87.0)	Improved functional capacity with effect maintained postsurgery	<0.001	Exercise and nutrition synergize for enhanced recovery and reduced decline in functional capacity.
Liu et al. 2020 [52]	33208327	China	526 surgical patients (37.5% male, 62.5% female; average age 53.47 y)	Temperature accuracy, peak temperature, and fever detection	iThermonitor WT705 compared with mercury thermometer	Perioperative period	526 (iThermonitor)	Accuracy: 0.03°C ± 0.35°C, fever peak: 37.55°C ± 0.59°C	526 (mercury thermometer)	Accuracy: –0.03°C ± 0.35°C, fever peak: 37.26°C ± 0.56°C	Fever detection: +4.35 h earlier (*P* < 0.001)	*P* < 0.001	iThermonitor demonstrated better fever detection, peak temperature measurement, and earlier fever identification.
Ausania et al. 2019 [53]	31232076	Spain	40 patients with pancreatic or periampullary tumors	Postoperative complications, delayed gastric emptying (DGE)	Standard care + prehabilitation compared with standard care alone	7–30 d before surgery	18	DGE: 5.6%	22	DGE: 40.9%	Relative risk reduction for DGE: 86%	*P* = 0.01 for DGE	Prehabilitation reduced delayed gastric emptying but did not significantly affect overall postoperative complications.
Gao et al., 2023 [54]	37456818	China	Adults awaiting elective cardiac surgery	6-min walk distance	Multimodal (exercise, nutrition, mindfulness)	1 wk presurgery	76	Improvement ≥20 m	76	No significant change	Improvement in functional capacity (6MWD)	<0.05	Prehabilitation enhanced functional capacity and psychological readiness in cardiac surgery patients.
Weindelmayer et al. 2021 [55]	33988796	Italy	Patients undergoing gastrectomy for cancer	Length of hospital stay, costs	ERAS protocol	Pre- and postsurgery	248	6 d (median)	103	8 d (median)	LOS reduced by 2 d, costs reduced by €1097	<0.001	ERAS improved recovery and reduced costs without increasing complications or readmissions.
Durán Poveda et al. 2023 [6]	37513700	Spain	469 patients with gastrointestinal cancer undergoing elective surgery	Nutritional risk via MUST and GLIM, postoperative complications	Nutritional screening and therapy	Admission to discharge	231	Nutritional therapy reduced malnutrition prevalence	237	High risk of malnutrition persisted in 47%	Moderate improvement in risk scores (p < 0.001)	*P* < 0.001	Screening and nutritional support reduce malnutrition risk but do not completely mitigate it during hospitalization.
Clemente-Suárez et al. 2022 [14]	35457471	Italy	Patients with cancer and cachexia	Muscle mass retention, functional mobility	Nutritional and exercise interventions	Presurgery	48	+4.0 kg muscle (1.2)	46	–2.0 kg muscle (0.9)	+6.0 (5.1–6.9)	<0.001	Interventions preserve muscle mass effectively in patients with cachexia.
Williams et al. 2019 [8]	30248745	United States	Malnourished surgical patients	Nutritional status, postoperative infections	Perioperative nutritional support	Pre- and post-op	120	10% infection rate (3%)	110	22% infection rate (5%)	–12% (–14 to –10%)	<0.001	Nutritional support reduces infection rates significantly.

Details the outcomes of various prehabilitation studies, including their population characteristics, intervention types, and outcome measurements. Key interventions include nutritional optimization, exercise regimens, and psychological support.

Abbreviations: 6MWT, 6-min Walk Test; ADL, Activities of Daily Living; APAIS, Amsterdam Preoperative Anxiety and Information Scale; CI, Confidence Interval; ERAS, Enhanced Recovery after Surgery; ET, Endurance Time; GLIM, Global Leadership Initiative on Malnutrition; ICU, Intensive Care Unit; KOOS, Knee Injury and Osteoarthritis Outcome Score; LOS, length of stay; MUST, Malnutrition Universal Screening Tool; QOL, Quality of Life; RCT, Randomized Controlled Trial; RR, Relative Risk; TKA, Total Knee Arthroplasty; VAT, Ventilatory Anaerobic Threshold ; VO_2_, oxygen uptake.

Evidence supports the role of nutritional prehabilitation in improving surgical outcomes, including reducing the length of hospital stays and readmissions. A systematic review of patients undergoing elective colorectal surgeries found that those receiving nutritional prehabilitation were discharged an average of 2 d earlier than control groups [64]. This reduction in hospital stay was attributed to faster recovery of gut function, reduced complications, and improved wound healing, facilitated by better nutritional status. Similarly, a recent meta-analysis evaluating the effects of prehabilitation in gastrointestinal surgeries reported a significant reduction in overall complications, particularly infectious ones, in patients receiving nutritional interventions [65,66]. However, it is challenging to attribute these reductions in hospital stay solely to prehabilitation, as the implementation and refinement of enhanced recovery after surgery protocols during the period of the systematic review likely introduced a bias against observing a difference between prehabilitation and control groups. The effect of nutritional prehabilitation also varies depending on the type of surgery and patient population. For instance, patients undergoing liver resections experienced a pronounced reduction in complications, whereas those undergoing low-risk laparoscopic procedures showed less substantial benefits [67]. This variability underscores the need for tailored prehabilitation protocols to optimize outcomes based on patient and surgical factors.

In cancer surgeries, the interplay between neoadjuvant therapies and prehabilitation has been a focus of research. Neoadjuvant chemotherapy and radiotherapy are known to exacerbate malnutrition and muscle loss, increasing the risk of surgical complications [68]. Studies have shown that prehabilitation during this period can counteract these effects. For instance, patients receiving prehabilitation although undergoing neoadjuvant therapy for rectal cancer experienced improved nutritional status and fewer complications compared with controls [69]. Regardless of these promising findings, some studies have reported varying results, particularly in long-term outcomes such as quality of life and survival. Although a systematic review evaluating nutritional prehabilitation in major abdominal surgeries found no significant difference in mortality rates between the intervention and control groups [58], it is important to note that postoperative mortality is not necessarily a long-term outcome, as deaths occurring shortly after surgery are included in such measures. Oncological outcomes, such as 5-y survival and disability-free survival, are often overlooked but are critically important, especially in the context of cancer surgery. These endpoints provide a more comprehensive assessment of the long-term efficacy of prehabilitation programs. This limitation highlights a broader critique of many prehabilitation trials, which often focus on short-term metrics and fail to address outcomes that hold greater relevance for long-term recovery and functional independence. Future studies should prioritize these endpoints to better elucidate the true value of prehabilitation in improving patient trajectories.

## Multimodal Integration of Nutritional Prehabilitation

The success of prehabilitation lies in its multidisciplinary nature, wherein nutritional interventions are integrated with exercise and psychological support. This synergistic approach addresses multiple facets of patient vulnerability, enhancing physiological and psychological resilience before surgery (Table 1). The interplay between these modalities amplifies the benefits of each, making multimodal prehabilitation an essential strategy for optimizing surgical outcomes.

### The role of exercise in nutritional prehabilitation

The objective of prehabilitation is not solely to preserve muscle mass but to build a preoperative physiological reserve. This proactive approach prepares patients to better withstand the catabolic challenges of the postoperative period and recovery, ultimately enhancing their ability to return to baseline function or better. Physical training complements nutritional interventions by improving the body’s ability to utilize energy sources efficiently, increasing cardiovascular fitness, and preserving muscle mass. This proactive approach anticipates the catabolic state that patients experience postoperatively and during recovery, better equipping them to withstand surgical stress and achieve a faster return to baseline function. Exercise stimulates anabolic pathways, promoting protein synthesis and optimizing the utilization of nutrients provided during nutritional prehabilitation (Figure 1). This is particularly relevant for patients with diminished physical reserves, such as those with sarcopenia or chronic illnesses [70]. Aerobic exercise (AE) is a cornerstone of prehabilitation, significantly enhancing cardiovascular capacity, which is critical for mitigating the metabolic demands of surgery. AE improves oxygen delivery and utilization, which is critical for surgical recovery and tissue repair [71,72]. Resistance training [resistance exercise (RE)], on the other hand, targets skeletal muscle, counteracting the catabolic effects of surgery and malnutrition. A study by Mikami et al. [73] demonstrated that a combination of AE and RE in pancreatic cancer patients led to significant improvements in peak oxygen uptake (VO_2_ peak), a marker of cardiopulmonary fitness, during the prehabilitation period. Combined with high-protein diets or amino acid supplementation, exercise can significantly reduce muscle atrophy and improve postoperative functional capacity. For example, studies have demonstrated that patients participating in multimodal prehabilitation programs exhibit greater improvements in mobility and strength compared with those receiving unimodal interventions [74]. Improved VO_2_ peak correlates with a reduced risk of postoperative complications, particularly in surgeries requiring prolonged anesthesia and recovery periods. In a further analysis by Van Wijk et al. [49], patients with liver and pancreatic cancer undergoing prehabilitation with high-intensity interval training showed marked improvements in their aerobic capacity compared with those receiving standard care. These findings highlight the potential of structured aerobic training to enhance preoperative fitness and postoperative recovery.

Resistance training, often combined with aerobic components, is integral to preserving and enhancing skeletal muscle mass and strength during prehabilitation. A RCT by Ausania et al. [53] observed that multimodal prehabilitation, which included RE and nutritional support, resulted in significant gains in hand grip strength and improved performance in functional mobility tests. These benefits are particularly vital for patients prone to sarcopenia conditions that increase susceptibility to surgical complications. Furthermore, Parker et al. [48] demonstrated that exercise-based prehabilitation in patients with pancreatic cancer preserved skeletal muscle mass and prevented the decline observed in control groups. Muscle preservation through resistance training also aids in reducing fatigue and accelerating postoperative recovery. Exercise exerts anti-inflammatory effects, complementing the benefits of nutritional optimization. Studies have shown that exercise-induced modulation of inflammatory pathways reduces levels of proinflammatory cytokines, such as IL-6 and TNF-α [75]. This anti-inflammatory response mitigates the systemic inflammation associated with surgical stress. The incorporation of mobility exercises, such as walking programs and stretching routines, during prehabilitation enhances functional capacity and supports early mobilization postsurgery. A systematic review by Coulshed et al. [76] highlighted that exercise-based prehabilitation significantly improved functional metrics; including the 6MWT and timed-up-and-go (TUG) tests. Although these preoperative functional improvements are promising, evidence linking them directly to shorter hospital stays or major postoperative outcomes remains unpredictable. This underscores the need for further investigation into the relationship between preoperative functional gains and postoperative recovery trajectories.

### Psychological support and nutritional adherence

The psychological preparation of patients undergoing surgery is a critical, yet often overlooked, component of prehabilitation. Adherence can be challenging, especially for patients with anxiety, depression, or low self-efficacy. Studies have shown that psychological support can address these barriers effectively. For instance, Gillis et al. [45] demonstrated that a multimodal prehabilitation program incorporating psychological education and counseling improved patient compliance with prescribed high-protein diets and immunonutrition. By mitigating anxiety and fostering a sense of control, these interventions enable patients to adhere more consistently to their nutritional plans. Another trial by Barberan-Garcia et al. [44] reported that patients receiving psychological support in combination with dietary counseling showed higher adherence to protein and caloric intake recommendations compared with those without such support. Relaxation techniques, such as guided imagery and mindfulness training, have proven effective in reducing anxiety levels and improving focus on prehabilitation goals. Minnella et al. [51] highlighted that patients participating in relaxation-based psychological interventions were more likely to meet their nutritional and exercise targets, leading to better functional recovery postsurgery.

Psychological education sessions provide patients with information about the benefits of nutritional and physical prehabilitation, empowering them to make informed decisions. Hirst et al. [77] found that patients who understood the rationale behind nutritional interventions were more likely to adhere to dietary guidelines. Group-based cognitive behavioral therapy (CBT), employed in some trials, has also been shown to improve adherence rates. By addressing maladaptive thoughts and behaviors, CBT helps patients build resilience and stay committed to their prehabilitation programs [78]. The integration of psychological support with nutritional and physical interventions enhances the overall effectiveness of prehabilitation. A systematic review by Alsuwaylihi et al. [79] found that patients participating in multimodal programs with a psychological component demonstrated better adherence to dietary and exercise protocols than those in unimodal or bimodal programs.

### Timing and personalization of multimodal prehabilitation

The timing of multimodal prehabilitation is crucial for maximizing its benefits. Initiating interventions within a timeframe of 4–8 wk before surgery strikes an ideal balance between program adherence and efficacy, provided the underlying disease permits. Durations shorter than 2–4 wk are largely ineffective, whereas prehabilitation extending beyond 3 mo often results in poor patient adherence. However, in urgent surgical cases, even a shorter prehabilitation period can yield measurable benefits, emphasizing the importance of flexibility in program design [80]. Personalization is a cornerstone of effective multimodal prehabilitation. Nutritional strategies should be tailored to the patient’s baseline metabolic status, surgical risk, and specific nutritional deficits. Although screening for nutritional deficiencies in the preoperative period is ideal, implementing universal laboratory testing for all patients may not be feasible because of logistical and financial constraints, particularly on a large scale. A pragmatic approach may involve targeted screening based on clinical risk factors, such as significant weight loss, low BMI, or symptoms suggestive of malnutrition (for example, fatigue, muscle weakness). Tools like the Nutritional Risk Screening (2002) or the Malnutrition Universal Screening Tool can help identify high-risk patients for more detailed assessment. Similarly, exercise regimens must align with individual fitness levels and limitations, including musculoskeletal or weight-bearing restrictions. Reference-standard assessments like cardiopulmonary exercise test (CPET) or VO_2_ max testing can guide aerobic training intensity (70%–80% threshold) but are costly and impractical at scale. Simpler alternatives like the 6MWT or TUG test offer actionable insights for tailoring programs. Resistance training can be scaled using wearable devices to monitor adherence and adjust intensity remotely. For patients with physical restrictions, low-impact or aquatic exercises can maintain safe engagement. Future trials should stratify participants by baseline fitness and employ dynamic adjustments to optimize outcomes. Technology integration and hybrid models can make personalization scalable. Psychological interventions addressing surgery-related stress further enhance outcomes when integrated into multimodal prehabilitation, emphasizing the importance of a holistic, adaptable framework (Table 2).

**TABLE 2:** Summary of prehabilitation strategies and outcomes across phases.

Weeks	Preoperative	Postoperative	Operation	Technique	Initial Level	Progress	Actions	Homework
Preparation Phase								
4–6 weeks	Nutritional assessment: calorie and protein needs; initiation of immunonutrition	Education on postoperative recovery nutrition and exercise	Counseling on surgical risks and expected outcomes	Diet consultations; physical training plans	Baseline nutritional status assessed through BMI and serum albumin levels	Personalized diet plans with incremental protein and micronutrient supplementation	Weekly check-ins with dietitian to adjust intake	Maintain food diaries; follow prescribed nutritional supplements
	Functional evaluation: 6-min walk test and strength assessments	Baseline functional level logged for comparison	Physical preparation through resistance and aerobic exercises	Supervised resistance and cardio sessions	Functional deficits identified through tests like stair climbing	Strength improvements recorded every two weeks	Engage with physiotherapists for feedback	Perform prescribed strength and endurance exercises at home daily
	Psychological preparation: CBT and mindfulness	Identifying mental health triggers for surgical anxiety	Preparing emotionally for recovery process	Relaxation and stress management techniques	Baseline anxiety levels assessed using standardized scales	Reduction in anxiety levels tracked weekly	Scheduled consultations with psychological support staff	Practice relaxation and mindfulness techniques daily
	Introduction to wearable devices for tracking progress	Postoperative tracking guidance	Review importance of patient adherence	Hands-on wearable device tutorials	Baseline data collection (e.g., steps, heart rate)	Weekly data analysis by healthcare team	Provide feedback on adherence barriers	Track physical activity and sleep patterns through devices
	Introduction to telemedicine platform	Orientation on virtual consultations	Emergency protocol reminders	Online introductory consultations	Assess patient familiarity with telemedicine	Smooth transition into virtual monitoring	Set up and test connections for sessions	Log queries and challenges faced for team review
Active Prehabilitation Phase								
2–4 weeks	Implementation of high-protein and micronutrient-dense diets	Monitoring tolerance to prescribed diets	Optimizing energy stores for operation	High-calorie, highprotein meal plan	Weight and intake compliance measured weekly	Incremental weight gain or stabilization	Adjust caloric content based on weekly progress	Adhere strictly to meal plans and report difficulties
	Structured physical training: alternating resistance and aerobic exercises	Addressing postoperative fatigue through lowintensity activities	Reducing muscle loss during recovery	Gym-based resistance training; walking programs	Baseline exercise capacity logged (distance, reps)	Increased endurance and strength monitored bi-weekly	Attend physiotherapy sessions as scheduled	Perform supplementary home exercises thrice weekly
	Continued psychological support	Focus on postoperative mental health preparation	Establishing realistic recovery expectations	Regular CBT sessions	Psychological triggers reassessed through weekly scales	Decline in anxiety and improved focus	Follow-up calls to ensure mental readiness	Engage in daily relaxation practices
	Tracking wearable device data	Begin postoperative tracking setup	Final data check before surgery	Analyzing progress with devices	Weekly updates on tracked metrics	Maintain stable or improved metrics presurgery	Align expectations with collected data	Use tracked data to selfregulate physical activity levels
Post-Surgery Phase								
1–2 weeks	Reinforcement of dietary compliance	Encouragement to transition to recovery diets	Monitoring initial healing response	Light, digestible protein meals	Digestive tolerance assessed post-surgery	Incremental calorie intake progression	Regular dietitian reviews	Follow soft diet progression charts
	Resumption of low-intensity physical activity	Increase stamina and rebuild strength	Short, guided walks or breathing exercises	Short walking circuits and chair exercises	Initial steps and repetitions recorded	Gradual improvement logged weekly	Collaborate with physiotherapists for adjustments	Perform light stretches and short walks daily
	Emotional recovery support	Focus on building postsurgery resilience	Providing mental health resources	Tele-counseling for recovery stress	Postoperative anxiety reassessed through scales	Steady improvement in mental state	Bi-weekly virtual check-ins	Record and share challenges faced during recovery
	Telemedicine platform integration	Weekly check-ins to evaluate progress	Ensuring full recovery postsurgery	Remote monitoring sessions	Initial postoperative metrics uploaded for review	Improved metrics monitored weekly	Participate in guided follow-up activities	Log self-assessment observations for follow-up sessions

The table provides an overview of prehabilitation parameters, capturing the intervention timeline, type, and associated outcomes across pre- and postsurgical phases.

Abbreviation: CBT, cognitive behavioral therapy.

## The Role of Emerging Technologies in Prehabilitation

Emerging technologies have revolutionized the delivery of prehabilitation programs, addressing traditional barriers such as accessibility, scalability, and patient adherence. By incorporating wearable devices, telemedicine platforms, mobile applications, and AI, prehabilitation programs can now offer more personalized and efficient interventions. These innovations have proven particularly useful during the COVID-19 pandemic, where remote healthcare delivery became essential.

### Wearable devices and real-time monitoring

Wearable devices equipped with sensors to track physical activity, heart rate, and sleep patterns are becoming integral tools in prehabilitation. By offering real-time data, these devices allow healthcare providers to customize interventions based on individual needs. Moreover, they foster patient motivation by providing tangible feedback on progress. Finley et al. [81] demonstrated that patients using wrist-worn smartwatches during prehabilitation showed significant improvements in adherence to exercise protocols, resulting in enhanced functional capacity measured through the 6MWT. Similarly, Poirier et al. [82] found that activity trackers improved adherence to prescribed walking regimens by 25%, leading to an average 50-m increase in walking distance compared with controls. Amin et al. [83] further reported that wearable devices reduced preoperative anxiety by fostering a sense of control and readiness, which translated to faster postoperative recovery. Some studies report a significant number of participants reported difficulties with the consistent use of wearable devices, posing a considerable challenge to the effectiveness of remote prehabilitation programs. Although telemedicine oversight has demonstrated promise in enhancing compliance and improving outcomes by fostering greater patient accountability [84], accessibility remains a critical issue. Individuals who are wealthier, younger, and more educated are often better positioned to adopt and utilize these technologies, exacerbating disparities in health outcomes. Patients from lower socioeconomic backgrounds or those with limited technological proficiency may find these tools less accessible, further widening the gap in who benefits from these interventions. Addressing these disparities requires a focus on equitable solutions. Simplified device interfaces, subsidized access to technology, and community-based support systems are critical strategies to ensure broader participation. Hybrid approaches that blend remote technologies with periodic in-person interactions may also help mitigate these issues, offering a more inclusive and effective model. Despite the potential of remote prehabilitation, studies often report diminished efficacy compared with supervised programs, primarily because of challenges in monitoring compliance and effort. Without real-time feedback and supervision, patients may not complete prescribed training regimens with the required intensity, limiting the intervention's benefits. Although wearable devices and AI provide tools for tracking compliance, they cannot fully replicate the motivational and corrective advantages of direct supervision. To optimize prehabilitation programs, future research must directly compare the outcomes of supervised and remote models. A systematic evaluation of hybrid approaches that combine the accessibility of remote technology with intermittent in-person sessions could offer a balanced solution.

### Telemedicine and remote prehabilitation

Telemedicine platforms have become critical for delivering multimodal prehabilitation, enabling patients to access nutritional counseling, exercise training, and psychological support remotely. These platforms eliminate logistical barriers like travel and clinic availability, making them particularly useful in rural or underserved areas. A systematic review by Tay et al. [85] highlighted that telemedicine-delivered prehabilitation programs achieved outcomes comparable with traditional in-person interventions, with notable reductions in preoperative anxiety and improved functional capacity. Remote monitoring via video conferencing and data-sharing features ensures continuity of care although providing convenience. Piraux et al. [86] compared telemedicine-based prehabilitation with in-person programs for colorectal surgery patients and found similar improvements in VO_2_ peak and functional capacity. The telemedicine group also reported higher satisfaction because of the convenience of virtual consultations. Nevertheless, the hybrid model of exercise supervision could provide a more flexible and inclusive approach to prehabilitation. In such a framework, remote prehabilitation would serve as the default mode of delivery, leveraging telemedicine and mobile technologies to reach a broader population. Patients identified as noncompliant, facing challenges with exercise instructions, or requiring medical supervision for participation in strenuous exercise could then transition to supervised in-person sessions. This adaptive approach ensures that individuals with unique needs or difficulties receive appropriate support although maintaining the accessibility and scalability advantages of remote programs. The hybrid model also holds promise for optimizing resource allocation, as in-person supervision is reserved for those who need it most. Additionally, research should evaluate the cost-effectiveness and feasibility of hybrid models, particularly in resource-limited settings, to ensure their practicality and broader implementation.

### AI and personalized prehabilitation

AI-driven tools have transformed prehabilitation by enabling highly personalized interventions based on patient-specific data. These systems analyze factors such as genetic, metabolic, and behavioral information to tailor nutritional and exercise plans. AI algorithms can predict responses to dietary changes and identify optimal exercise regimens, thereby maximizing the benefits of prehabilitation. Yoon et al. [87] report in a review that AI-based risk stratification improved resource allocation in prehabilitation programs, prioritizing intensive interventions for high-risk patients. This approach resulted in a 20% reduction in postoperative complications compared with standard care. Additionally, predictive analytics facilitated by AI track adherence trends and flag early signs of declining engagement, allowing healthcare teams to intervene proactively. Virtual reality (VR) has emerged as a promising tool in prehabilitation, providing immersive environments for both relaxation and exercise. A study by Schrempf et al. [88] demonstrated that VR-based relaxation significantly improved mood and well-being in patients undergoing colorectal cancer surgery, with high adherence rates and minimal adverse effects. Similarly, a pilot study by Aburrous et al. [89] explored the use of VR exercise games in prehabilitation for bariatric surgery patients. Although compliance with prescribed exercise regimens remained a challenge, VR-based interventions showed the potential to enhance engagement through interactive and gamified environments. In the context of exercise prehabilitation, VR offers the flexibility of remote engagement although maintaining patient motivation. The web-based exercise program described by Bennell et al. [90] exemplifies how individualized, technology-assisted interventions can improve adherence and functional outcomes during the perioperative period. These findings suggest that VR could complement traditional prehabilitation by providing an engaging and accessible platform for exercise, particularly for patients with limited mobility or access to in-person programs. Future research should evaluate the scalability and efficacy of VR-enabled prehabilitation compared with traditional supervised approaches. Incorporating tailored VR interventions that address physical and psychological needs could bridge gaps in preoperative care, offering a viable alternative for diverse patient populations.

## Implementation Challenges and Future Directions

Despite the growing recognition of prehabilitation as a transformative approach in surgical care, its widespread implementation faces significant challenges. These barriers include resource limitations, variability in healthcare settings, a lack of standardized protocols, and issues with patient adherence. Addressing these challenges is critical to realizing the full potential of prehabilitation in enhancing surgical outcomes and improving patient resilience.

### Resource constraints in healthcare settings

One of the primary obstacles to implementing prehabilitation programs is the high demand for resources. Comprehensive prehabilitation often requires the coordinated efforts of dietitians, physiotherapists, psychologists, and healthcare administrators. Many healthcare institutions, especially in low-resource settings, struggle to allocate these resources, limiting the availability of prehabilitation programs [91]. Additionally, the cost of specialized nutritional supplements, exercise facilities, and monitoring equipment can be prohibitive for patients and healthcare systems alike. To address resource constraints, scalable models of prehabilitation are essential. Simplified protocols leveraging community-based services can effectively reduce the burden on hospitals although maintaining accessibility. For example, partnerships with local gyms or fitness centers could provide patients with supervised exercise sessions, reducing the reliance on hospital-based resources. Similarly, nutrition counseling could be outsourced to community dietitians or wellness centers, ensuring patients receive tailored guidance without straining clinical staff. Public health policies that incentivize preventive care could further drive the adoption of prehabilitation. For instance, government-subsidized programs for exercise or nutrition counseling could be expanded to include prehabilitation. Additionally, workplace wellness programs could integrate prehabilitation services for employees undergoing elective surgeries, creating a cost-sharing model that benefits both employers and healthcare systems.

### Variability in healthcare systems

The implementation of prehabilitation varies widely across healthcare systems, reflecting differences in infrastructure, patient demographics, and clinical priorities. In some regions, access to advanced surgical and perioperative care is limited, making it challenging to introduce prehabilitation programs. Even in well-resourced settings, inconsistencies in program design and delivery hinder the establishment of evidence-based practices. This variability complicates efforts to standardize prehabilitation protocols and evaluate their effectiveness across diverse populations [92]. To overcome these issues, a global effort is needed to establish guidelines for prehabilitation. International collaborations, facilitated by professional organizations and research networks, can play a pivotal role in developing and disseminating these standards. Training healthcare professionals in the principles and practice of prehabilitation is also essential to ensure its consistent and effective application.

### Lack of standardized protocols

The heterogeneity of prehabilitation programs poses a significant challenge to their implementation and evaluation. Differences in the duration, intensity, and components of prehabilitation interventions make it difficult to compare outcomes across studies and establish best practices [93]. For instance, some programs focus solely on nutritional supplementation, whereas others adopt a multimodal approach that includes exercise and psychological support. The absence of standardized outcome measures further complicates efforts to assess the efficacy of prehabilitation. Developing standardized protocols requires a consensus among researchers and clinicians on key elements of prehabilitation. These elements should include criteria for patient selection, specific nutritional and exercise interventions, and measurable outcomes such as functional capacity, complication rates, and quality of life. Standardization would facilitate the pooling of data from multiple studies, enabling meta-analyses that provide stronger evidence for the benefits of prehabilitation. Furthermore, the prospect of undergoing surgery can itself be a source of anxiety, which may negatively impact patients’ ability to participate actively in prehabilitation. To enhance patient adherence, prehabilitation programs must be designed with a patient-centered approach. This involves tailoring interventions to individual needs, preferences, and capabilities. Personalized care plans that account for patients’ baseline fitness levels, nutritional status, and psychological profiles are more likely to be effective. Additionally, providing patients with clear information about the benefits of prehabilitation and offering regular support from healthcare providers can boost motivation and engagement.

### The way forward: future directions

Future research should focus on addressing the gaps in knowledge and practice that currently limit the effectiveness of prehabilitation. Long-term studies are needed to evaluate the sustained impact of prehabilitation on recovery, quality of life, and healthcare costs. These studies should include diverse patient populations to ensure that findings are generalizable across different settings and demographics. High-risk populations, such as the elderly, malnourished, and those with chronic illnesses, should be prioritized in prehabilitation research. Understanding the unique needs and challenges of these groups will inform the development of targeted interventions that maximize their benefits. Additionally, exploring the integration of prehabilitation into existing surgical pathways and enhanced recovery programs will help establish its role as a standard component of perioperative care. Collaboration among researchers, clinicians, policymakers, and technology developers is essential to advance the field of prehabilitation. By working together, these stakeholders can overcome the challenges of implementation and harness the opportunities offered by innovation. Ultimately, prehabilitation has the potential to transform surgical care, empowering patients and improving outcomes on a global scale.

### Conclusion

Prehabilitation represents a transformative approach to surgical care, prioritizing proactive optimization of patients’ physical, nutritional, and psychological health. Among its components, nutritional prehabilitation plays a pivotal role in enhancing metabolic resilience, preserving muscle mass, and supporting immune function. By addressing the unique challenges posed by surgical stress, malnutrition, and comorbidities, nutritional strategies help patients withstand the demands of surgery and recover more effectively. The physiological mechanisms underlying nutritional prehabilitation, including its effects on insulin sensitivity, inflammation, and protein synthesis, underscore its critical importance in perioperative care. Clinical evidence supports its benefits in reducing complications, improving functional outcomes, and accelerating recovery, particularly when integrated into multimodal prehabilitation frameworks. However, the variability in study designs and outcomes highlights the need for standardized protocols and further research. Tailoring nutritional interventions to specific populations, such as the elderly, patients with cancer, and those with metabolic disorders, ensures that prehabilitation addresses the diverse needs of surgical patients. Emerging technologies, including wearable devices, telemedicine, and AI, are enhancing the accessibility and personalization of prehabilitation, paving the way for more efficient and effective programs. Despite its promise, the widespread implementation of prehabilitation faces challenges, including resource constraints and the need for systemic changes in surgical pathways. Overcoming these barriers requires collaboration among multidisciplinary teams, investment in innovative solutions, and a commitment to patient-centered care. As prehabilitation continues to evolve, it has the potential to redefine the surgical experience, transforming it from a reactive process to a proactive journey of empowerment and resilience.

## Author contributions

The authors’ responsibilities were as follows – SSP: responsible for conceptualization, methodology, data analysis, and manuscript writing; DG: contributed equally through conceptualization, literature review, and drafting the original manuscript; DS: served as the lead author, provided supervision, and was responsible for conceptualization, methodology, data analysis, manuscript writing, and serving as the corresponding author; and all authors: read and approved the final manuscript.

## Data availability

Data will be made available on request.

## Funding

The authors reported no funding received for this study.

## Conflict of interest

The authors report no conflicts of interest.

## References

[1] Pinto A., Faiz O., Davis R., Almoudaris A., Vincent C. (2016). Surgical complications and their impact on patients' psychosocial well-being: a systematic review and meta-analysis. BMJ Open.

[2] Dobson G.P. (2020). Trauma of major surgery: a global problem that is not going away. Int. J. Surg..

[3] Reza T., Grezenko H., Barker C., Bakht D., Faran N., Abdullah Yahya N. (2023). Emotional stress and immune response in surgery: a psychoneuroimmunological perspective. Cureus.

[4] Bougeard A.M., Watkins B. (2019). Transitions of care in the perioperative period—a review. Clin. Med. (Lond)..

[5] Shakya P., Poudel S. (2022). Prehabilitation in patients before major surgery: a review article, JNMA J. Nepal. Med. Assoc..

[6] Durán Poveda M., Suárez-de-la-Rica A., Cancer Minchot E., Ocón Bretón J., Sánchez Pernaute A., Rodríguez Caravaca G. (2023). The prevalence and impact of nutritional risk and malnutrition in gastrointestinal surgical oncology patients: a prospective, observational, multicenter, and exploratory study. Nutrients.

[7] Martínez-Ortega A.J., Piñar-Gutiérrez A., Serrano-Aguayo P., González-Navarro I., Remón-Ruíz P.J., Pereira-Cunill J.L. (2022). Perioperative nutritional support: a review of current literature. Nutrients.

[8] Williams D.G.A., Molinger J., Wischmeyer P.E. (2019). The malnourished surgery patient: a silent epidemic in perioperative outcomes?. Curr. Opin. Anaesthesiol..

[9] Kutnik P., Wichowska O., Sysiak-Sławecka J., Szczukocka M., Rypulak E., Piwowarczyk P. (2023). Malnutrition risk in elective surgery patients and effectiveness of preoperative nutritional interventions at a pre-anaesthetic clinic: a 4-year apart, single-centre, observational study, Anaesthesiol. Intensive Ther..

[10] Wobith M., Hill A., Fischer M., Weimann A. (2024). Nutritional prehabilitation in patients undergoing abdominal surgery-a narrative review. Nutrients.

[11] Javed H., Olanrewaju O.A., Ansah Owusu F., Saleem A., Pavani P., Tariq H. (2023). Challenges and solutions in postoperative complications: a narrative review in general surgery. Cureus.

[12] Finnerty C.C., Mabvuure N.T., Ali A., Kozar R.A., Herndon D.N. (2013). The surgically induced stress response, JPEN J. Parenter. Enteral Nutr..

[13] Wernerman J., Christopher K.B., Annane D., Casaer M.P., Coopersmith C.M., Deane A.M. (2019). Metabolic support in the critically ill: a consensus of 19. Crit. Care..

[14] Clemente-Suárez V.J., Redondo-Flórez L., Rubio-Zarapuz A., Martínez-Guardado I., Navarro-Jiménez E., Tornero-Aguilera J.F. (2022). Nutritional and exercise interventions in cancer-related cachexia: an extensive narrative review. Int. J. Environ. Res. Public Health..

[15] van der Velde M., van der Leeden M., Geleijn E., Veenhof C., Valkenet K. (2023). What moves patients to participate in prehabilitation before major surgery? A mixed methods systematic review. Int. J. Behav. Nutr. Phys. Act..

[16] Banasiewicz T., Kobiela J., Cwaliński J., Spychalski P., Przybylska P., Kornacka K. (2023). Recommendations on the use of prehabilitation, i.e. comprehensive preparation of the patient for surgery. Pol. Przegl. Chir..

[17] Jones L., Tan L., Carey-Jones S., Riddell N., Davies R., Brownsdon A. (2021). Can wearable technology be used to approximate cardiopulmonary exercise testing metrics? Perioper. Med. (Lond)..

[18] Greco M., Angelucci A., Avidano G., Marelli G., Canali S., Aceto R. (2023). Wearable health technology for preoperative risk assessment in elderly patients: the WELCOME study. Diagnostics (Basel).

[19] Kneuertz P.J., Jagadesh N., Perkins A., Fitzgerald M., Moffatt-Bruce S.D., Merritt R.E. (2020). Improving patient engagement, adherence, and satisfaction in lung cancer surgery with implementation of a mobile device platform for patient-reported outcomes. J. Thorac. Dis..

[20] Geer E.B., Islam J., Buettner C. (2014). Mechanisms of glucocorticoid-induced insulin resistance: focus on adipose tissue function and lipid metabolism. Endocrinol. Metab. Clin. North Am..

[21] Beaupere C., Liboz A., Fève B., Blondeau B., Guillemain G. (2021). Molecular mechanisms of glucocorticoid-induced insulin resistance. Int. J. Mol. Sci..

[22] Bilku D.K., Dennison A.R., Hall T.C., Metcalfe M.S., Garcea G. (2014). Role of preoperative carbohydrate loading: a systematic review. Ann. R. Coll. Surg. Engl..

[23] Zhang H., Dhalla N.S. (2024). The role of pro-inflammatory cytokines in the pathogenesis of cardiovascular disease. Int. J. Mol. Sci..

[24] Slim K., Badon F., Vacheron C.H., Occean B.V., Dziri C., Chambrier C. (2022). Umbrella review of the efficacy of perioperative immunonutrition in visceral surgery. Clin. Nutr. ESPEN..

[25] Ahmed R., Augustine R., Chaudhry M., Akhtar U.A., Zahid A.A., Tariq M. (2022). Nitric oxide-releasing biomaterials for promoting wound healing in impaired diabetic wounds: state of the art and recent trends. Biomed. Pharmacother..

[26] Wu G., Meininger C.J., McNeal C.J., Bazer F.W., Rhoads J. (2021). Role of L-arginine in nitric oxide synthesis and health in humans. Adv. Exp. Med. Biol..

[27] Fischer R., Konkel A., Mehling H., Blossey K., Gapelyuk A., Wessel N. (2014). Dietary omega-3 fatty acids modulate the eicosanoid profile in man primarily via the cyp-epoxygenase pathway. J. Lipid. Res..

[28] Padoan F., Piccoli E., Pietrobelli A., Moreno L.A., Piacentini G., Pecoraro L. (2024). The role of zinc in developed countries in pediatric patients: a 360-degree view. Biomolecules.

[29] Prasad A.S., Bao B. (2019). Molecular mechanisms of zinc as a pro-antioxidant mediator: clinical therapeutic implications. Antioxidants (Basel).

[30] Piccirillo R., Demontis F., Perrimon N., Goldberg A.L. (2014). Mechanisms of muscle growth and atrophy in mammals and drosophila. Dev. Dyn..

[31] Workeneh B., Bajaj M. (2013). The regulation of muscle protein turnover in diabetes. Int. J. Biochem. Cell Biol..

[32] Kaspy M.S., Hannaian S.J., Bell Z.W., Churchward-Venne T.A. (2024). The effects of branched-chain amino acids on muscle protein synthesis, muscle protein breakdown and associated molecular signalling responses in humans: an update. Nutr. Res. Rev.

[33] Dimou A., Tsimihodimos V., Bairaktari E. (2022). The critical role of the branched chain amino acids (BCAAs) catabolism-regulating enzymes, branched-chain aminotransferase (BCAT) and branched-chain α-keto acid dehydrogenase (BCKD), in human pathophysiology. Int. J. Mol. Sci..

[34] Gwin J.A., Church D.D., Allen J.T., Wilson M.A., Carrigan C.T., Murphy N.E. (2025). Consuming whey protein with added essential amino acids, not carbohydrate, maintains postexercise anabolism while underfed. Med. Sci. Sports. Exerc..

[35] Cava E., Padua E., Campaci D., Bernardi M., Muthanna F.M.S., Caprio M. (2024). Investigating the health implications of whey protein consumption: a narrative review of risks, adverse effects, and associated health issues. Healthcare (Basel).

[36] Kumar M., Leon Coria A., Cornick S., Petri B., Mayengbam S., Jijon H.B. (2020). Increased intestinal permeability exacerbates sepsis through reduced hepatic scd-1 activity and dysregulated iron recycling. Nat. Commun..

[37] Stolfi C., Maresca C., Monteleone G., Laudisi F. (2022). Implication of intestinal barrier dysfunction in gut dysbiosis and diseases. Biomedicines.

[38] You S., Ma Y., Yan B., Pei W., Wu Q., Ding C. (2022). The promotion mechanism of prebiotics for probiotics: a review. Front. Nutr..

[39] Roy S., Dhaneshwar S. (2023). Role of prebiotics, probiotics, and synbiotics in management of inflammatory bowel disease: current perspectives. World J. Gastroenterol..

[40] Rastgoo S., Ebrahimi-Daryani N., Agah S., Karimi S., Taher M., RashidKhani B. (2021). Glutamine supplementation enhances the effects of a low FODMAP diet in irritable bowel syndrome management. Front. Nutr..

[41] Wang G., Yang F., Zhou W., Xiao N., Luo M., Tang Z. (2023). The initiation of oxidative stress and therapeutic strategies in wound healing. Biomed. Pharmacother..

[42] Tappel A.L. (1980). Vitamin E and selenium protection from in vivo lipid peroxidation. Ann. N. Y. Acad. Sci..

[43] De Pasquale G., Mancin S., Matteucci S., Cattani D., Pastore M., Franzese C. (2023). Nutritional prehabilitation in head and neck cancer: a systematic review of literature. Clin. Nutr. ESPEN..

[44] Barberan-Garcia A., Ubré M., Roca J., Lacy A., Burgos F., Risco R. (2018). Personalised prehabilitation in high-risk patients undergoing elective major abdominal surgery: a randomized blinded controlled trial. Ann. Surg..

[45] Gillis C., Loiselle S.-E., Fiore J.F., Awasthi R., Wykes L., Liberman A.S. (2016). Prehabilitation with whey protein supplementation on perioperative functional exercise capacity in patients undergoing colorectal resection for cancer: a pilot double-blinded randomized placebo-controlled trial. J. Acad. Nutr. Diet..

[46] Waller E., Sutton P., Rahman S., Allen J., Saxton J., Aziz O. (2022). Prehabilitation with wearables versus standard of care before major abdominal cancer surgery: a randomised controlled pilot study (trial registration: NCT04047524). Surg. Endosc..

[47] Correia F.D., Nogueira A., Magalhães I., Guimarães J., Moreira M., Barradas I. (2019). Medium-term outcomes of digital versus conventional home-based rehabilitation after total knee arthroplasty: prospective, parallel-group feasibility study, JMIR Rehabil. Assist. Technol..

[48] Parker N., Gorzelitz J., Ngo-Huang A., Caan B., Prakash L., Garg N. (2021). The role of home-based exercise in maintaining skeletal muscle during preoperative pancreatic cancer treatment, Integr. Cancer Ther.

[49] van Wijk L., Bongers B.C., Berkel A.E.M., Buis C.I., Reudink M., Liem M.S.L. (2022). Improved preoperative aerobic fitness following a home-based bimodal prehabilitation programme in high-risk patients scheduled for liver or pancreatic resection. Br. J. Surg..

[50] Chiu P.L., Li H., Yap K.Y.-L., Lam K.C., Yip P.R., Wong C.L. (2023). Virtual reality-based intervention to reduce preoperative anxiety in adults undergoing elective surgery: a randomized clinical trial. JAMA Netw. Open.

[51] Minnella E.M., Awasthi R., Loiselle S.-E., Agnihotram R.V., Ferri L.E., Carli F. (2018). Effect of exercise and nutrition prehabilitation on functional capacity in esophagogastric cancer surgery: a randomized clinical trial. JAMA Surg.

[52] Liu Y., Liu C., Gao M., Wang Y., Bai Y., Xu R. (2020). Evaluation of a wearable wireless device with artificial intelligence, iThermonitor WT705, for continuous temperature monitoring for patients in surgical wards: a prospective comparative study. BMJ Open.

[53] Ausania F., Senra P., Meléndez R., Caballeiro R., Ouviña R., Casal-Núñez E. (2019). Prehabilitation in patients undergoing pancreaticoduodenectomy: a randomized controlled trial. Rev. Esp. Enferm. Dig..

[54] Gao W., Li H., Chen Y., Zhang Y., Zhang M., Jin J. (2023). Effectiveness of a short-term multimodal prehabilitation program in adult patients awaiting selective cardiac surgery: study protocol for an open-label, pilot, randomized controlled trial. Front. Cardiovasc. Med..

[55] Weindelmayer J., Mengardo V., Gasparini A., Sacco M., Torroni L., Carlini M. (2021). Enhanced recovery after surgery can improve patient outcomes and reduce hospital cost of gastrectomy for cancer in the west: a propensity-score-based analysis. Ann. Surg. Oncol..

[56] Jain S.N., Lamture Y., Krishna M. (2023). Enhanced recovery after surgery: exploring the advances and strategies. Cureus.

[57] Loon M.M., Goshe M., Rashid M., Shehryar A., Rehman A., Abdallah S. (2024). Impact of preoperative nutritional support on surgical outcomes in gastrointestinal surgeries: a systematic review. Cureus.

[58] McIsaac D.I., Gill M., Boland L., Hutton B., Branje K., Shaw J. (2022). Prehabilitation in adult patients undergoing surgery: an umbrella review of systematic reviews. Br. J. Anaesth..

[59] Wu D., Lewis E.D., Pae M., Meydani S.N. (2018). Nutritional modulation of immune function: analysis of evidence, mechanisms, and clinical relevance. Front. Immunol..

[60] Yu J., Yuan A., Liu Q., Wang W., Sun Y., Li Z. (2024). Effect of preoperative immunonutrition on postoperative short-term clinical outcomes in patients with gastric cancer cachexia: a prospective randomized controlled trial. World J. Surg. Oncol..

[61] Halliday L.J., Boshier P.R., Doganay E., Wynter-Blyth V., Buckley J.P., Moorthy K. (2023). The effects of prehabilitation on body composition in patients undergoing multimodal therapy for esophageal cancer. Dis. Esophagus..

[62] Walsh M., Martindale R. (2024). A review of perioperative immune-modulating and metabolic-modulating nutrition strategies for bowel resection surgery. JPEN J. Parenter. Enteral. Nutr..

[63] Daniels S.L., Lee M.J., George J., Kerr K., Moug S., Wilson T.R. (2020). Prehabilitation in elective abdominal cancer surgery in older patients: systematic review and meta-analysis. BJS Open.

[64] Zhong J.X., Kang K., Shu X.L. (2015). Effect of nutritional support on clinical outcomes in perioperative malnourished patients: a meta-analysis. Asia Pac. J. Clin. Nutr..

[65] Jain S.R., Kandarpa V.L., Yaow C.Y.L., Tan W.J., Ho L.M.L., Sivarajah S.S. (2023). The role and effect of multimodal prehabilitation before major abdominal surgery: a systemic review and meta-analysis. World J. Surg..

[66] Mareschal J., Hemmer A., Douissard J., Dupertuis Y.M., Collet T.H., Koessler T. (2023). Surgical prehabilitation in patients with gastrointestinal cancers: impact of unimodal and multimodal programs on postoperative outcomes and prospects for new therapeutic strategies-a systematic review. Cancers (Basel).

[67] Stephanos M., Stewart C.M.B., Mahmood A., Brown C., Hajibandeh S., Hajibandeh S. (2024). Low versus standard central venous pressure during laparoscopic liver resection: a systematic review, meta-analysis and trial sequential analysis. Ann. Hepatobiliary Pancreat. Surg..

[68] Xu X.Y., Jiang X.M., Xu Q., Xu H., Luo J.H., Yao C. (2022). Skeletal muscle change during neoadjuvant therapy and its impact on prognosis in patients with gastrointestinal cancers: a systematic review and meta-analysis. Front. Oncol..

[69] Guerra-Londono C.E., Cata J.P., Nowak K., Gottumukkala V. (2024). Prehabilitation in adults undergoing cancer surgery: a comprehensive review on rationale, methodology, and measures of effectiveness. Curr. Oncol..

[70] Dickinson J.M., Volpi E., Rasmussen B.B. (2013). Exercise and nutrition to target protein synthesis impairments in aging skeletal muscle, Exerc. Sport Sci. Rev..

[71] Doyle M.P., Indraratna P., Tardo D.T., Peeceeyen S.C., Peoples G.E. (2019). Safety and efficacy of aerobic exercise commenced early after cardiac surgery: a systematic review and meta-analysis. Eur. J. Prev. Cardiol..

[72] Cuijpers A.C.M., Bongers B.C., Heldens A.F.J.M., Bours M.J.L., van Meeteren N.L.U., Stassen L.P.S. (2022). Aerobic fitness and muscle density play a vital role in postoperative complications in colorectal cancer surgery. J. Surg. Oncol..

[73] Mikami Y., Kouda K., Kawasaki S., Okada K.-I., Kawai M., Kitahata Y. (2020). Preoperative in-hospital rehabilitation improves physical function in patients with pancreatic cancer scheduled for surgery. Tohoku J. Exp. Med..

[74] Toohey K., Hunter M., McKinnon K., Casey T., Turner M., Taylor S. (2023). A systematic review of multimodal prehabilitation in breast cancer. Breast Cancer Res. Treat..

[75] Gómez-Rubio P., Trapero I. (2019). The effects of exercise on IL-6 levels and cognitive performance in patients with schizophrenia. Diseases.

[76] Coulshed A., Coulshed D., Pathan F. (2023). Systematic review of the use of the 6-minute walk test in measuring and improving prognosis in patients with ischemic heart disease. CJC Open.

[77] Hirst Y., Lim A.W.W. (2018). Acceptability of text messages for safety netting patients with low-risk cancer symptoms: a qualitative study. Br. J. Gen. Pract..

[78] Curtiss J.E., Levine D.S., Ander I., Baker A.W. (2021). Cognitive-behavioral treatments for anxiety and stress-related disorders. Focus (Am. Psychiatr. Publ.)..

[79] Alsuwaylihi A., Skořepa P., Prado C.M., Gomez D., Lobo D.N., O'Connor D. (2024). Exploring the acceptability of and adherence to prehabilitation and rehabilitation in patients undergoing major abdominal surgery: a systematic review and meta-analysis. Clin. Nutr. ESPEN..

[80] Bates A., West M.A., Jack S., Grocott M.P.W. (2024). Preparing for and not waiting for surgery. Curr. Oncol..

[81] Finley D.J., Fay K.A., Batsis J.A., Stevens C.J., Sacks O.A., Darabos C. (2020). A feasibility study of an unsupervised, pre-operative exercise program for adults with lung cancer. Eur. J. Cancer Care..

[82] Poirier J., Bennett W., Jerome G., Shah N., Lazo M., Yeh H.-C. (2016). Effectiveness of an activity tracker- and internet-based adaptive walking program for adults: a randomized controlled trial. J. Med. Internet. Res..

[83] Amin T., Mobbs R.J., Mostafa N., Sy L.W., Choy W.J. (2021). Wearable devices for patient monitoring in the early postoperative period: a literature review. mHealth.

[84] Kang H.S., Exworthy M. (2022). Wearing the future-wearables to empower users to take greater responsibility for their health and care: scoping review, JMIR Mhealth. Uhealth..

[85] Tay S.S., Zhang F., Neo E.J.R. (2024). The use of technology in cancer prehabilitation: a systematic review. Front. Oncol..

[86] Piraux E., Caty G., Reychler G., Forget P., Deswysen Y. (2020). Feasibility and preliminary effectiveness of a tele-prehabilitation program in esophagogastric cancer patients. J. Clin. Med..

[87] Yoon H.K., Yang H.L., Jung C.W., Lee H.C. (2022). Artificial intelligence in perioperative medicine: a narrative review. Korean J. Anesthesiol..

[88] Schrempf M.C., Zanker J., Arndt T.T., Vlasenko D., Anthuber M., Müller G. (2023). Immersive virtual reality fitness games to improve recovery after colorectal surgery: a randomized single blind controlled pilot trial. Games Health J.

[89] Aburrous M., Adwan H., Tam A., Gendia A., Cota A., Finlay I. (2023). Can virtual reality exercise games improve compliance with preoperative prescriptive exercise programs in patients awaiting bariatric surgery?. Br. J. Surg..

[90] Bennell K.L., Nelligan R., Dobson F., Rini C., Keefe F., Kasza J. (2019). Does a web-based exercise programming system improve home exercise adherence for people with musculoskeletal conditions? A randomized controlled trial. Am. J. Phys. Med. Rehabil..

[91] Miller L.J., Halliday V., Snowden J.A., Aithal G.P., Lee J., Greenfield D.M. (2024). Health professional attitudes and perceptions of prehabilitation and nutrition before haematopoietic cell transplantation. J. Hum. Nutr. Diet..

[92] Heil T.C., Driessen E.J.M., Argillander T.E., Melis R.J.F., Maas H.A.A.M., Olde Rikkert M.G.M. (2022). Implementation of prehabilitation in colorectal cancer surgery: qualitative research on how to strengthen facilitators and overcome barriers. Support Care Cancer.

[93] Skořepa P., Ford K.L., Alsuwaylihi A., O'Connor D., Prado C.M., Gomez D. (2024). The impact of prehabilitation on outcomes in frail and high-risk patients undergoing major abdominal surgery: a systematic review and meta-analysis. Clin. Nutr..

